# Mycotic Hepatic Artery Aneurysm in Staphylococcus aureus Infective Endocarditis

**DOI:** 10.7759/cureus.96333

**Published:** 2025-11-07

**Authors:** Adnan Ahmed, Ayesha Javaid, Rosica Panayotova

**Affiliations:** 1 Cardiology, Stockport NHS Foundation Trust, Manchester, GBR; 2 Cardiology, Countess of Chester, Chester, GBR

**Keywords:** endocarditis, hepatic artery anuerysm, infection, mycotic, staph aureus endocarditis

## Abstract

We present the case of a woman in her 70s with a history of rheumatoid arthritis on long-term immunosuppression, previous breast cancer, and interstitial lung disease, who was admitted with fever, rigors, malaise, and gastrointestinal symptoms. Blood cultures grew *Staphylococcus aureus*, and echocardiography revealed a large aortic valve vegetation, confirming infective endocarditis. Later imaging identified discitis, bilateral renal infarcts, and a 3 cm hepatic artery aneurysm. During the course of treatment, she developed severe aortic regurgitation. She underwent hepatic artery embolization followed by tissue aortic valve replacement, and completed six weeks of intravenous antibiotics. At three-month follow-up, she was asymptomatic and had returned to normal daily activities. This case highlights the potential for disseminated embolic complications of *S. aureus* infective endocarditis, particularly in immunosuppressed patients, and underscores the importance of prompt multidisciplinary intervention to optimize outcomes.

## Introduction

Staphylococcus aureus infective endocarditis remains potentially life-threatening infection associated with high rates of systemic embolization and complications. Up to 25% of patients with infective endocarditis demonstrate evidence of embolic phenomena at the time of diagnosis [1]. Among these, mycotic aneurysms represent a rare but serious manifestation, resulting from septic embolization and infectious seeding of the arterial wall [2]. A mycotic hepatic artery aneurysm arises due to infection-induced destruction of the arterial wall, leading to weakening and the formation of a blind, saccular outpouching [3]. This pathology carries a significant risk of rupture, reported in up to 14% of cases and associated with considerable morbidity and mortality [4]. Early recognition is critical, particularly in patients with known infective endocarditis presenting with new-onset abdominal pain or gastrointestinal symptoms [3]. We present a case of S. aureus infective endocarditis in an immunosuppressed woman complicated by hepatic artery aneurysm managed successfully through staged multidisciplinary intervention.

## Case presentation

A woman in her 70s presented to our Emergency Department with a one-week history of fever, rigors, and malaise, accompanied by diarrhoea and vomiting. Her past medical history included rheumatoid arthritis, for which she was on long-term immunosuppression, a history of breast cancer treated with surgery, chemotherapy and radiotherapy, and interstitial lung disease. Her regular medications on admission included leflunomide 20 mg daily, hydroxychloroquine 200 mg daily, atorvastatin 20 mg daily, and mirtazapine. The patient lived independently with her partner. She had a history of tobacco use but had since ceased smoking. She consumed alcohol socially and had no documented drug allergies.

On examination, her temperature was 38.9°C, heart rate was 114 bpm, and blood pressure was 104/60 mmHg, with oxygen saturation maintained on room air. Her initial physical examination was unremarkable. Cardiac ausculation revealed normal heart sounds. During the course of hospital stay, she developed small haemorrhagic macules on her palms and soles consistent with Janeway lesions.

Initial laboratory investigations revealed the following results (Table 1).

**Table 1:** Initial investigations

Parameter	Result	Reference Range
White cell count (WCC)	4.6 × 10⁹/L	4.0–11.0 × 10⁹/L
Haemoglobin (Hb)	107 g/L	115–155 g/L (female)
Mean corpuscular volume (MCV)	77 fL	80–100 fL
Platelet count	63 × 10⁹/L	150–400 × 10⁹/L
Neutrophils	3.9 × 10⁹/L	2.0–7.5 × 10⁹/L
Lymphocytes	0.5 × 10⁹/L	1.0–4.0 × 10⁹/L
Urea	6.7 mmol/L	2.5–7.8 mmol/L
Creatinine	98 μmol/L	45–90 μmol/L (female)
eGFR	50 mL/min/1.73 m²	> 60 mL/min/1.73 m²
Sodium (Na⁺)	128 mmol/L	135–145 mmol/L
Potassium (K⁺)	3.4 mmol/L	3.5–5.0 mmol/L
Alanine aminotransferase (ALT)	37 U/L	< 35 U/L (female)
Alkaline phosphatase (ALP)	88 U/L	30–130 U/L
Gamma-glutamyl transferase (GGT)	103 U/L	< 40 U/L (female)
Albumin	33 g/L	35–50 g/L
Prothrombin time (PT)	11.7 s	9.0–12.5 s
International normalised ratio (INR)	1	0.8–1.2
Glucose	6.8 mmol/L	3.5–7.8 mmol/L (random)
High-sensitivity troponin	< 1 ng/L	< 14 ng/L
pH (arterial)	7.47	7.35–7.45
pCO₂	3.8 kPa	4.7–6.0 kPa
pO₂	9.4 kPa	10.0–13.0 kPa (room air)
Bicarbonate (HCO₃⁻)	20.9 mmol/L	22–28 mmol/L
Base excess	−3.7	−2 to +2
Lactate	2.51 mmol/L	< 2.0 mmol/L
C-reactive protein (CRP)	322.3 mg/L	< 5 mg/L

**Figure 1:**
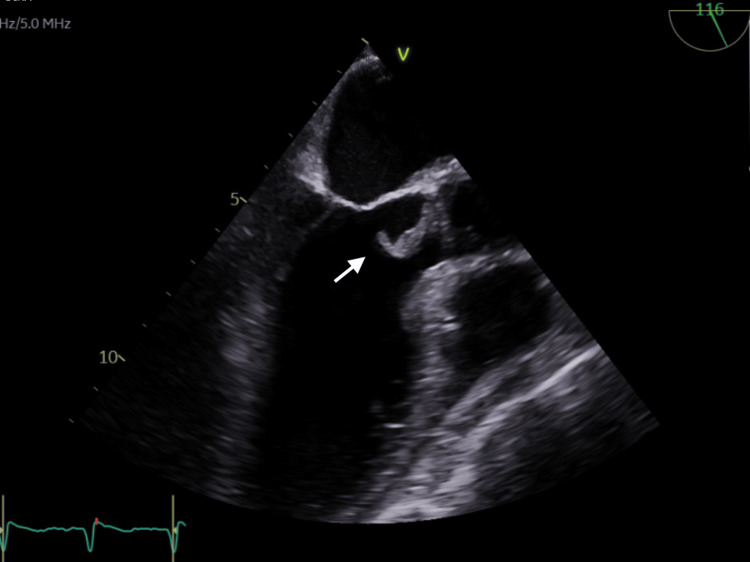
Transesophageal echocardiogram; mid-oesophageal long axis view in systole showing a 0.63 cm x 1.59 cm vegetation (white arrow) attached to the non-coronary cusp.

**Figure 2:**
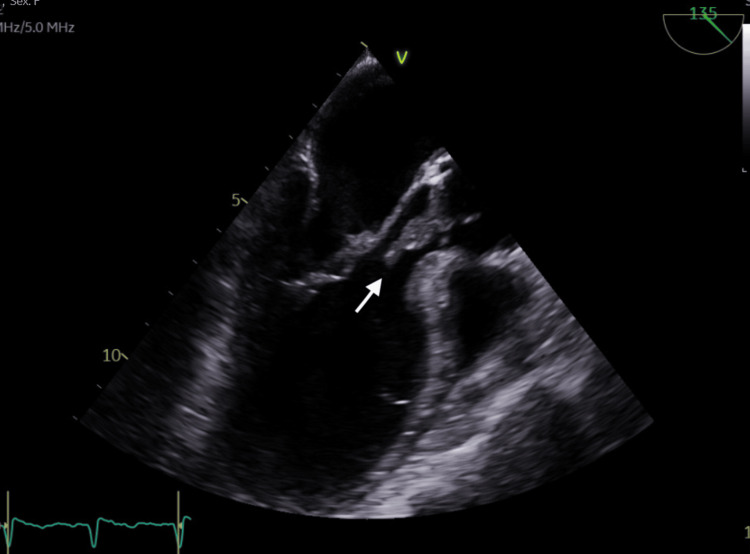
Transesophageal echocardiogram; mid-oesophageal long axis view in diastole showing a 0.63 cm x 1.59 cm vegetation (white arrow) attached to the non-coronary cusp.

Blood cultures were positive for Staphylococcus aureus (×3). A transthoracic echocardiogram revealed a mobile mass of 1.8 cm x 1 cm on the aortic valve projecting into the left ventricular outflow tract and left ventricular ejection fraction of 45%. Based on these findings, she was diagnosed with definite infective endocarditis according to the modified Duke Criteria. A transoesophageal echocardiogram performed showed a mobile vegetation (0.63 cm × 1.59 cm) on the non-coronary cusp, mild aortic regurgitation, and persistently reduced left ventricular systolic function (Figures 1, 2).

**Figure 3:**
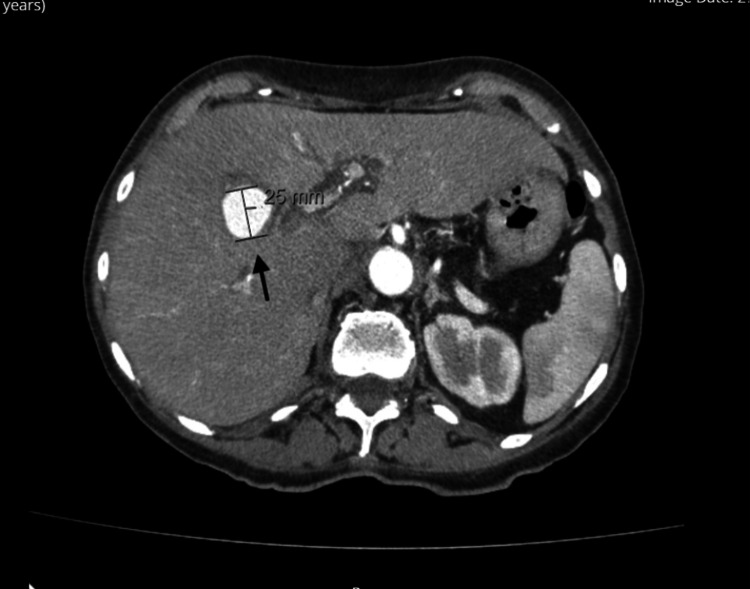
Triple phasic Computed Tomography of the liver in the axial view demonstrating a 3 cm x 2.5 cm aneurysm (black arrow) arising from a branch of the left hepatic artery.

**Figure 4:**
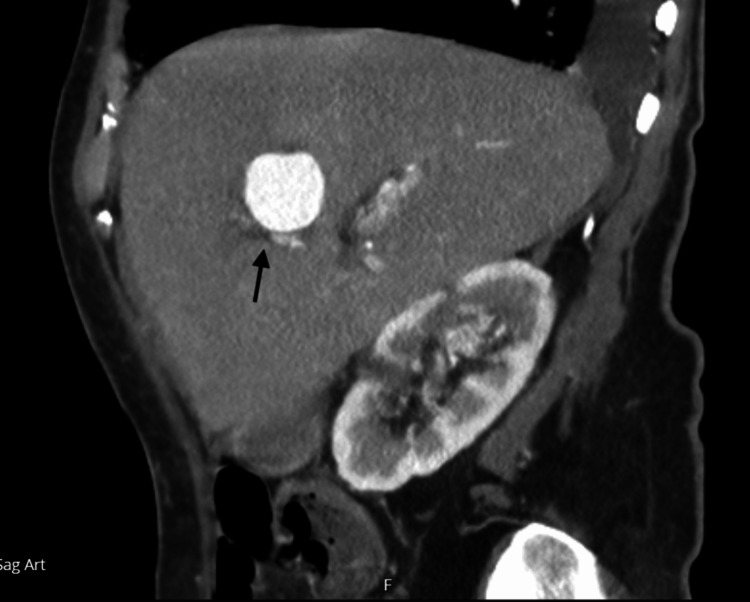
Triple phasic Computed Tomography of the liver in the sagittal view demonstrating a 3 cm x 2.5 cm aneurysm (black arrow) arising from a branch of the left hepatic

**Figure 5:**
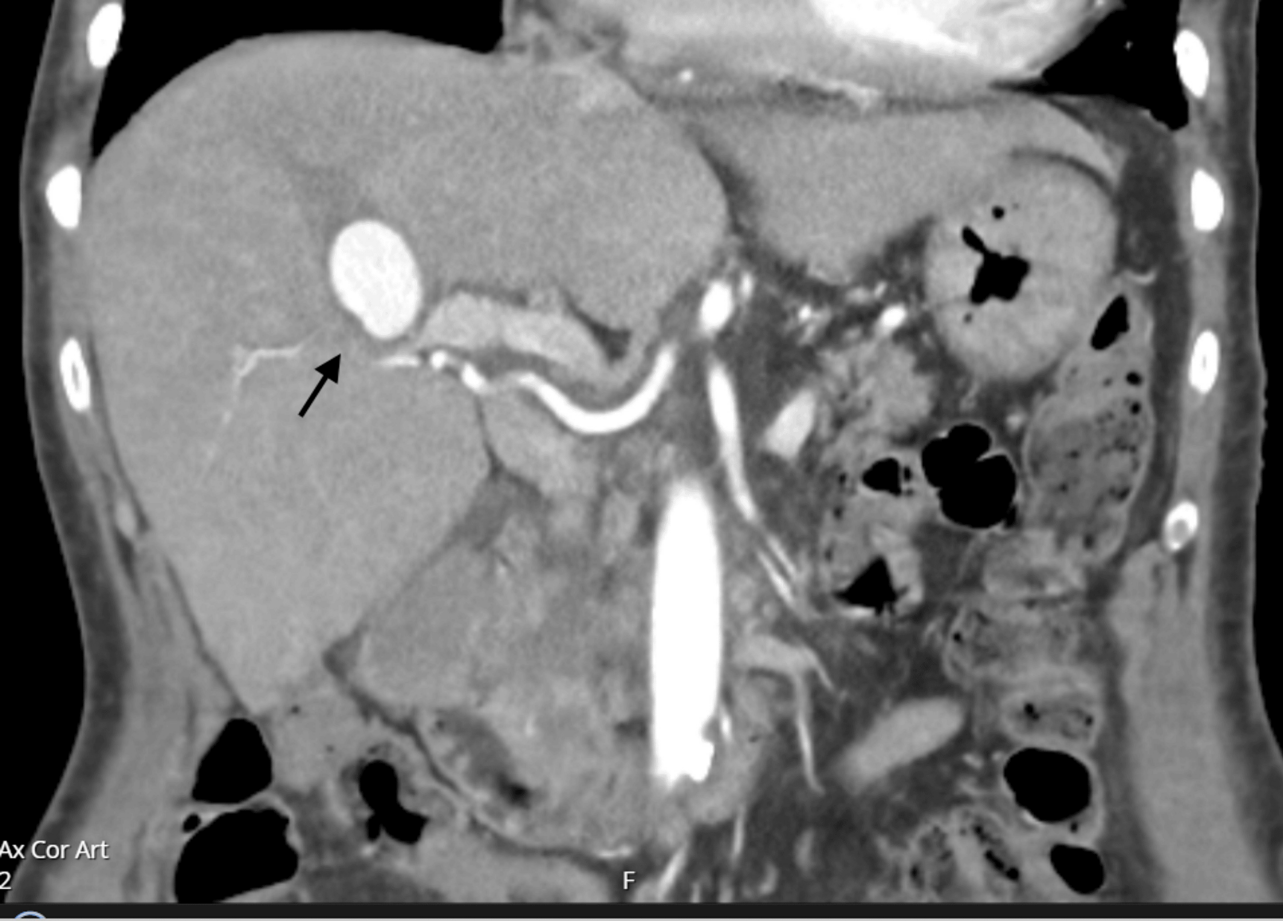
Triple phasic Computed Tomography of the liver in the coronal view identifying a 3 cm x 2.5 cm aneurysm (black arrow) arising from a branch of the left hepatic artery.

**Figure 6:**
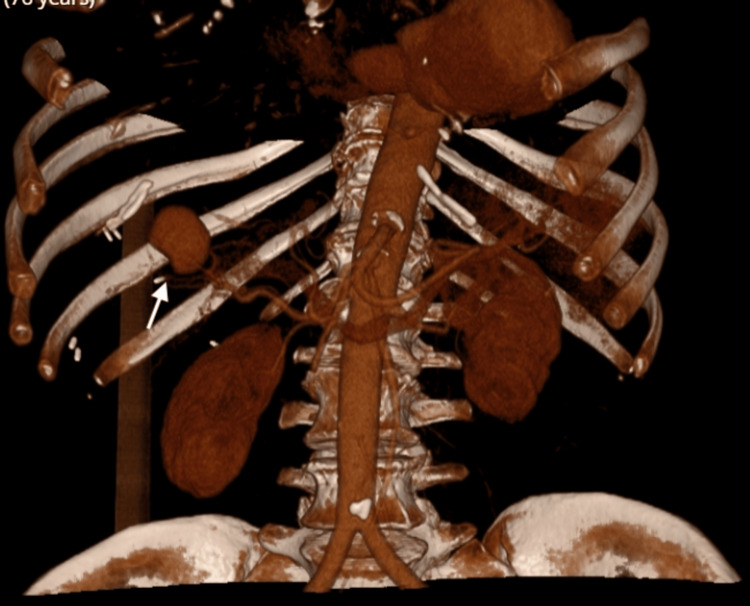
Spectral CT abdomen in the arterial phase shows a 3 cm × 2.5 cm aneurysm (white arrow) arising from a branch of the left hepatic artery consistent with a mycotic aneurysm.

Computed tomography of the abdomen and pelvis raised suspicion of T10-T11 discitis, which was subsequently confirmed by the spinal multidisciplinary team. The plan was to continue six weeks of IV antibiotics with a repeat CT spine. Her transthoracic echocardiogram after 16 days of antibiotic therapy revealed no change in the size of the vegetation. Three days prior to finishing her antibiotic regimen, she became acutely unwell with severe abdominal pain, nausea, and vomiting. Her cardiac auscultation revealed soft A2, and a grade III diastolic murmur best heard at the left lower sternal edge. A repeat CT abdomen revealed a 3 cm hyperdense lesion in the liver which appeared continuous with the anterior branch of right portal vein suspicious for pseudoaneurysm or hematoma, along with distal portal vein thrombosis. There were areas of patchy hypoattenuation in the kidneys bilaterally. A triple-phase CT liver identified a 3 cm × 2.5 cm aneurysm arising from a branch of the left hepatic artery (Figures 3-6).

A bedside echocardiogram performed the same day showed severe aortic regurgitation with a pressure half-time of 160 milliseconds and a large vegetation of 1.8 cm prolapsing into the left ventricular outflow tract.

Antibiotic therapy was initiated with intravenous teicoplanin, considering suspected methicillin-resistant Staphylococcus aureus, but later switched to IV flucloxacillin for four weeks after receiving drug sensitivity report when methicillin-sensitive Staphylococcus was confirmed. Leflunomide was withheld due to infection, and a cholestyramine washout was commenced, with plans to restart the medication after infection recovery. Given the presence of three distant embolic complications (kidneys, spine, and hepatic circulation), severe aortic regurgitation, and the high bleeding risk posed by the untreated hepatic artery aneurysm during cardiopulmonary bypass, clinical priority was given to embolization of the hepatic artery aneurysm before proceeding with cardiac surgery.

The patient underwent successful fluoroscopic hepatic artery embolization under interventional radiology. Post-procedure, the patient developed acute pulmonary oedema, which was managed medically with IV diuretics. She was then transferred to a tertiary cardiothoracic centre, where she underwent tissue aortic valve replacement. The patient had a favourable clinical recovery following these interventions with no immediate post-procedural complications. Intravenous antibiotics were continued for a total of six weeks, guided by microbiology and serial blood cultures. She remained haemodynamically stable throughout the remainder of her admission.

Cardiology follow-up was arranged for long-term management of valve disease and systolic dysfunction. At her three-month outpatient review, the patient was asymptomatic with no recurrence of abdominal pain. She had returned to full daily activities. She continues to be followed by cardiology.

## Discussion

Despite ongoing antibiotic therapy for Staphylococcus aureus infective endocarditis, our patient developed a sudden onset of acute abdominal pain, raising immediate concern for systemic embolization, a well-recognised complication due to the aggressive embolic potential of S. aureus. This prompted further diagnostic imaging.

This case shows a rare and complex presentation of Staphylococcus aureus infective endocarditis complicated by multiple systemic embolic events, including spinal discitis and a hepatic artery mycotic aneurysm, an unusual and potentially fatal manifestation. A systematic review and meta-analysis found that septic embolism occurs in approximately 25% of patients with infective endocarditis [5]. Several features are known to increase the risk of embolization, including vegetation size greater than 10 mm, high mobility, mitral valve involvement rather than aortic, and a C-reactive protein level above 40 mg/L [6]. The brain is the most frequently affected site, followed by solid organs such as the spleen, kidneys, and lungs [7]. Embolic events involving the spleen, kidneys, and certain areas of the brain are often clinically silent and are typically detected through routine imaging performed to assess for distant complications [8]. While embolic phenomena are well-documented in infective endocarditis, the simultaneous occurrence of discitis, visceral aneurysm, bilateral renal infarcts and severe aortic regurgitation from valvular destruction is infrequent and poses significant diagnostic and therapeutic challenges.

The hepatic artery aneurysm in our case likely represented a mycotic aneurysm secondary to septic embolization. Evidence on hepatic artery aneurysms associated with infective endocarditis (HAA-IE) is primarily derived from individual case reports and a single systematic review. In that review, which included a retrospective evaluation of patients with infective endocarditis across two centres, 10 cases of HAA-IE were identified among 623 patients, indicating a prevalence of 1.6% within the IE population [9]. All identified cases involved left-sided infective endocarditis, with the mitral valve being most affected (six mitral, one aortic, and three involving both valves) [9]. In a Mayo Clinic study involving 306 patients with visceral artery aneurysms, hepatic artery aneurysms accounted for 12% of cases, with only one instance attributed to infective endocarditis (0.3%) [4,10]. Diagnosis of the hepatic artery aneurysms in all cases was established through abdominal CT angiography [9]. Streptococcus species were the most isolated organisms followed by Staphylococcus [9]. Percutaneous coil embolization is considered the first-line treatment for symptomatic and asymptomatic HAA-IE of >15 mm in diameter [9]. In cases where rupture has occurred or embolization is not feasible, surgical intervention remains the definitive approach [11].

These infected HAAs have a high propensity to rupture [9], particularly in the context of anticoagulation or cardiopulmonary bypass, necessitating prompt recognition and intervention. In this patient, the decision to embolize the hepatic artery aneurysm before aortic valve surgery exemplifies the need for strategic prioritization based on individual risk. This approach was crucial in reducing the risk of intraoperative bleeding and enabled the safe completion of subsequent valve replacement surgery.

## Conclusions

This case illustrates a rare manifestation of Staphylococcus aureus infective endocarditis complicated by a mycotic hepatic artery aneurysm - a life-threatening complication that occurred despite appropriate antibiotic therapy. Timely recognition and embolization were crucial in preventing rupture and safely facilitating subsequent aortic valve replacement. The simultaneous occurrence of visceral aneurysm, spinal discitis, and renal embolic lesions underscores the aggressive embolic potential of S. aureus, particularly in immunosuppressed patients, and highlights that embolic complications may arise even with guideline-directed therapy. Clinicians should maintain a high index of suspicion for visceral embolic events in patients with persistent or atypical symptoms. Successful management requires coordinated multidisciplinary collaboration among microbiology, cardiology, interventional radiology, and cardiothoracic surgery teams.
